# Unc119 Protects from *Shigella* Infection by Inhibiting the Abl Family Kinases

**DOI:** 10.1371/journal.pone.0005211

**Published:** 2009-04-17

**Authors:** Ramarao Vepachedu, Zunayet Karim, Ojas Patel, Nicholas Goplen, Rafeul Alam

**Affiliations:** 1 National Jewish Health, Denver, Colorado, United States of America; 2 University of Colorado at Denver Health Sciences Center, Denver, Colorado, United States of America; University of Liverpool, United Kingdom

## Abstract

**Background:**

Bacteria engage cell surface receptors and intracellular signaling molecules to enter the cell. Unc119 is an adaptor protein, which interacts with receptors and tyrosine kinases. Its role in bacterial invasion of cells is unknown.

**Methodology/Principal Findings:**

We used biochemical, molecular and cell biology approaches to identify the binding partners of Unc119, and to study the effect of Unc119 on Abl family kinases and *Shigella* infection. We employed loss-of-function and gain-in-function approaches to study the effect of Unc119 in a mouse model of pulmonary shigellosis. Unc119 interacts with Abl family kinases and inhibits their kinase activity. As a consequence, it inhibits Crk phosphorylation, which is essential for *Shigella* infection. Unc119 co-localizes with Crk and *Shigella* in infected cells. *Shigella* infectivity increases in Unc119-deficient epithelial and macrophage cells. In a mouse model of shigellosis cell-permeable TAT-Unc119 inhibits *Shigella* infection. Conversely, Unc119 knockdown in vivo results in enhanced bacterial invasion and increased lethality. Unc119 is an inducible protein. Its expression is upregulated by probacteria and bacterial products such as lipopolysacharide and sodium butyrate. The latter inhibits *Shigella* infection in mouse lungs but is ineffective in Unc119 deficiency.

**Conclusions:**

Unc119 inhibits signaling pathways that are used by *Shigella* to enter the cell. As a consequence it provides partial but significant protection from *Shigella* infections. Unc119 induction in vivo boosts host defense against infections.

## Introduction

During the bacterial infection various proteins from the pathogen and the host participate in bacterial entry and cellular response [1]. Pathogens like *Shigella* induce their own uptake into mammalian cells [2]. *Shigella* delivers multiple virulence proteins into host cells through the type III secretion system (TSS) [3], [4]. One of these effector proteins is IpaB, which interacts with the mammalian cell surface receptor CD44 [5], [6]. Another efefctor, IpaA interacts with the integrin α5β1 [7]. The interaction of *Shigella* effectors with these host receptors is likely to facilitate the initial contact of the bacterium to the cell membrane, especially in the region of cholesterol-rich lipid rafts. At this stage the TSS proteins interact with cellular proteins to reorganize the cytoskeletal structures that results in their engulfment [8]–[10]. One of the crucial intracellular processes that play an essential role in bacterial uptake is actin polymerization [9]. Actin polymerization leads to membrane ruffling, which promotes bacterial internalization. Once internalized, *Shigella* utilizes actin tail to move about the cell. *Shigella* induces actin polymerization through multiple ways. *Shigella*-derived IcsA mimics activated-Cdc42 and directly activates N-WASP, which promotes actin polymerization [11]. Another effector IpgB1 mimics the host Rho G and activates Rac1 through the DOCK180-ELMO pathway [12]. Rac1 activates WAVE1 and WASP to induce actin polymerization [13]. The CD44-associated Src family kinases also promote actin focus formation [14], [15]. The Abl family kinases—Abl and Arg also become activated during the *Shigella* infection [16]. Both these kinases have an actin binding domain. Abl kinase phosphorylates N-WASP [16], which is an essential activator of Arp2/3. The latter directly induces actin polymerization. Both kinases phosphorylate the cytoskeletal regulator Crk [17]. Crk cooperates with cortactin to facilitate actin reorganization. Arg possesses actin-microtubule cross-linking activity and plays an important role in lamellopodia formation [18].

Unc119 is an adaptor molecule that has SH2- and SH3-binding motifs in addition to other protein-protein interaction motifs. Unc119 binds and activates Lyn and Hck kinases through its SH2- and SH3-binding motifs [19]. Unc119 plays a significant role in T cell signaling by activating Lck and Fyn [20]. Unc119 activates Fyn in fibroblasts and facilitates their differentiation into myofibroblasts [21]. As mentioned previously, Src promotes *Shigella* infection [14]. For this reason we asked if Unc119 played a role in *Shigella* infection. To our surprise Unc119 inhibited *Shigella* infection. It did so by inhibiting Abl family kinases and their substrate Crk. On the basis of these findings we believe Unc119 represents a novel inhibitory mechanism in *Shigella* infection.

## Results

### Unc119 inhibits *Shigella* infection

**Figure 1:**
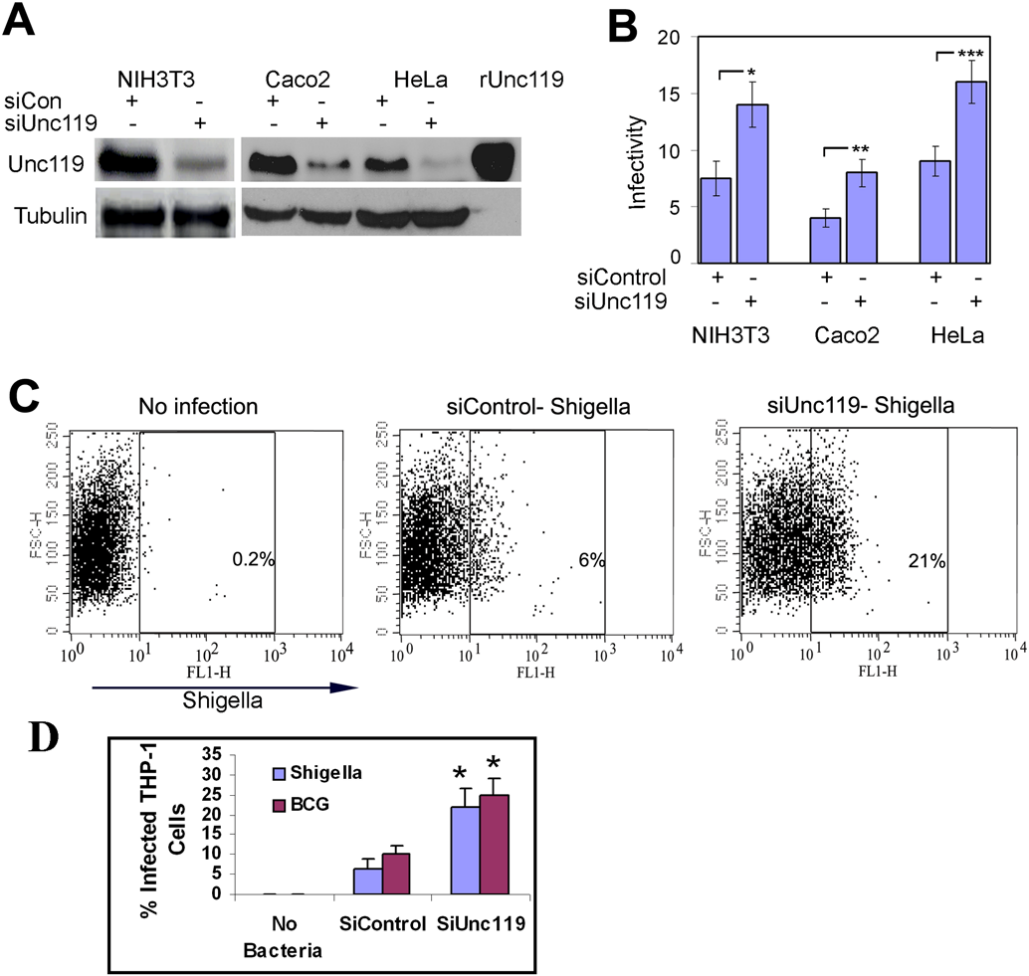
Unc119 inhibits *Shigella* infection. (A) Unc119 knockdown by siRNA. The expression of Unc119 in siUnc119- and control siRNA (siCon)-transfected cells was checked after 48 h by western blotting (N = 3). Equal loading was measured by reprobing the membrane for tubulin (lower panel). (B) Unc119 knockdown increases bacterial infection. 3T3, Caco2 and HeLa cells were transfected with siControl and siUnc119 RNA and then infected with *Shigella* flexneri. Following washes and gentamicin treatment the cells were lysed and plated for colony forming assay. The infectivity was expressed as the number of colony forming units per 10 cells. The results represent mean±SD of six independent experiments performed in triplicates. (*P = 0.012, **P = 0.014, ***P = 0.04). (C) Unc119 inhibition of *Shigella* infection in the THP-1 monocytic cell line. Preactivated THP-1 cells transfected with siRNA and then infected *Shigella* as described above. Fixed cells were stained with a rabbit anti-*Shigella* antibody and an Alexa 488 labeled secondary antibody. A representative flowgram is shown. (D) Unc119 inhibition of *Shigella* and BCG uptake by THP-1 cells. FITC-labeled Mycobacterium bovis (BCG) were used to study the effect of Unc119 knockdown (siUNc119 and SiControl) on bacterial uptake by THP-1 cells as measured by flow cytometry (see above and also Figure S1). Individual flow cytometry data for *Shigella* and BCG (N = 3 each) were analyzed statistically. *p<0.02.

Unc119 activates Src family kinases (SFKs) [19]–[21]. The latter facilitate bacterial internalization [14], [15]. We assumed that Unc119 would promote bacterial infection because of its role in SFK activation. To study the effect of Unc119 on bacterial infection, we knocked down Unc119 expression by 70–90% in human epithelial cells (Caco2 and HeLA) and mouse embryonic fibroblasts (NIH 3T3, abbreviated as 3T3) using siRNA (Figure 1A) and then infected them with *Shigella* flexneri. In all these cell lines the knockdown of Unc119 doubled the bacterial uptake (Figure 1B) as measured by a colony forming assay. A flow cytometric assay demonstrated a three-fold increase in *Shigella* uptake in Unc119 knocked-down THP-1 cells, a monocytic cell line (Figure 1C & D). The effect of Unc119 on *Shigella* infectivity was unaltered by aroA^−^ virG^−^ mutations that result in loss of intracellular movement of *Shigella* [22] (Figure S1A). The results suggest that Unc119 inhibition of infection is independent of the intracellular motility of *Shigella*. In order to determine whether Unc119 inhibits other bacterial infections, we studied Mycobacterium bovis BCG infection of THP-1 cells. Unc119 knockdown resulted in a two-fold increase in BCG uptake in THP-1 cells (Figure S1B and Figure 1D). The results suggest that the action of Unc119 is not specific for *Shigella*.

### Unc119 co-localizes with *Shigella*

**Figure 2:**
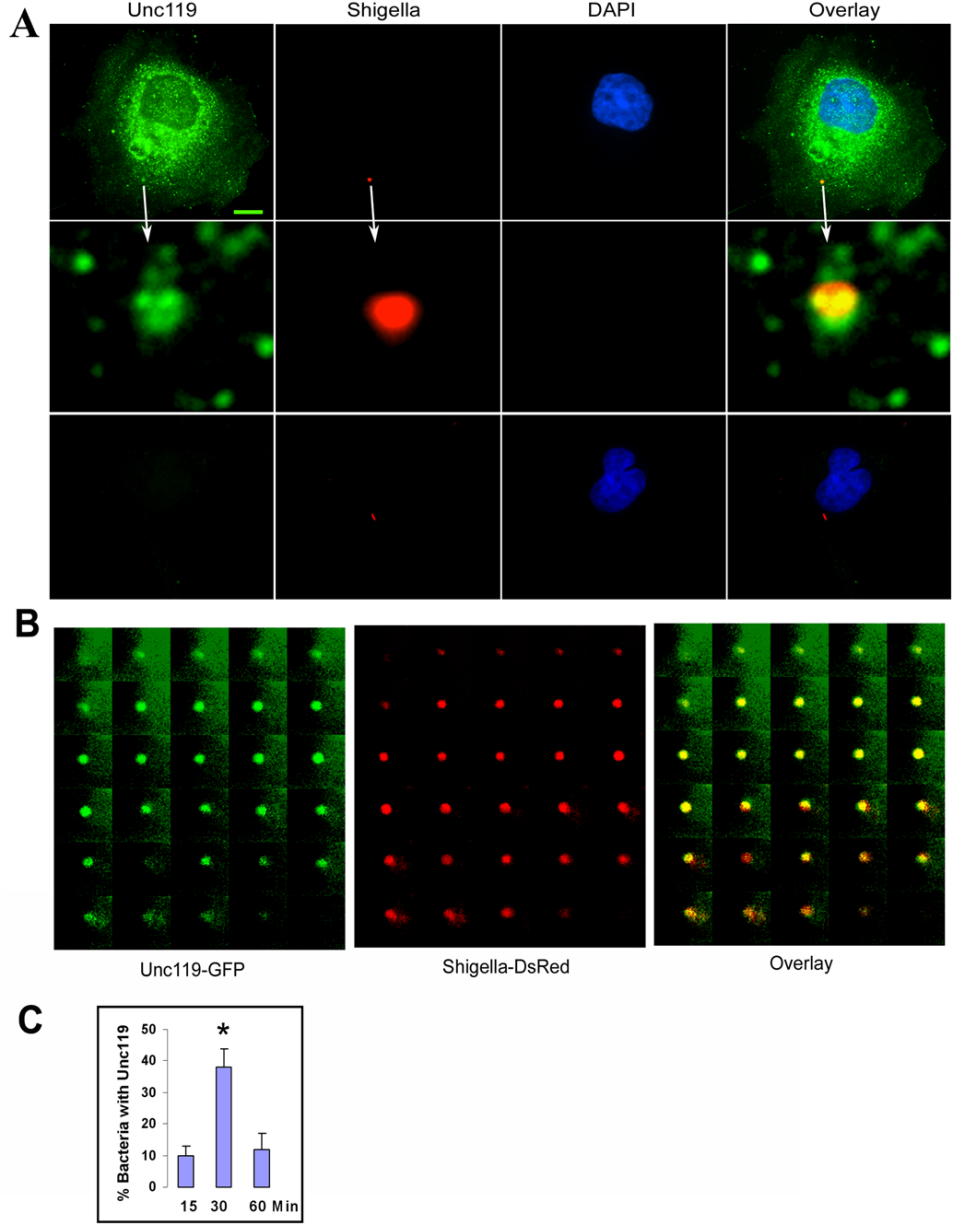
Interaction of Unc119 with *Shigella*. (A) Localization of Unc119 at the site of *Shigella* infection. HeLa cells were infected with *Shigella* expressing DsRed. The cells were stained for Unc119 using an affinity-purified anti-Unc119 rabbit antibody. The middle panel shows a close-up of the site of infection as indicated by an arrow. Lower panel shows staining with a rabbit IgG control antibody. A representative image from 3 independent experiments is shown. Scale bar, 5 µm. (B) Association of Unc119 with *Shigella* in live cells. Cells expressing GFP-Unc119 were infected with *Shigella* expressing DsRed. The cells were infected, then washed after 30 min of incubation and observed under microscope. The localization of Unc119 and *Shigella* was monitored using time-lapse video microscopy with 30 sec intervals for 15 min (N = 3). (C) Dynamics of Unc119 and *Shigella* association. The percent of bacteria that were associated with Unc119 is shown. 200 infected cells were counted per experiment (N = 3, *P<0.001).

In a next set of experiments HeLa cells were infected with DsRed expressing *Shigella* to study the localization of Unc119. Immunofluorescent staining showed a diffuse cytosolic distribution of Unc119 (Figure 2A). Unc119 was associated with vesicular structures in the cytosol and some of these vesicles were co-localized with the intracellular *Shigella* (Figure S1C). Next we examined the association of Unc119 with the internalized bacteria in live cells. Epifluorescence microscopy showed a time dependent association of Unc119-GFP with DsRed-expressing *Shigella*. Unc119-GFP remained associated with *Shigella* for up to 15 minutes (Figure 2B). The co-localization of Unc119-GFP with *Shigella* was highest at 30 minutes after the start of infection with 40% of the total internalized bacteria associated with Unc119-GFP (Figure 2C). The Unc119-*Shigella* association decreased to 10% by 60 minutes. We examined direct interaction of Unc119 with *Shigella*. Pull-down experiments with the *Shigella* lysate showed an interaction with 3 *Shigella*-derived proteins (Table S1), which may not be relevant as these proteins are not known to be secreted or expressed on the cell surface. At this time we do not know if Unc119 interacts with the type III secreted proteins of *Shigella*.

### Src family kinases do not mediate the effect of Unc119 on bacterial infection

CD44 and the α5β1 integrin are engaged by *Shigella* to enter into cells [5], [6]. Neutralization of CD44 and α5 integrin with antibodies inhibited *Shigella* uptake by 66% and 46% respectively (Figure S2A). When both receptors were neutralized the uptake was inhibited by 74%. Our results confirm that both CD44 and α5β1 are essential for bacterial uptake. Next, we explored the interaction of Unc119 with CD44 and α5β1. Immunoprecipitation studies showed that Unc119 precipitated with CD44 but not with α5 integrin (Figure S2B). The engagement of CD44 results in activation of Src [23], which influences infection [24]. Unc119 activates Lck, Hck and Fyn but not Src [19]. We explored the activation of Fyn, a ubiquitously expressed SFK [18], [19], during the bacterial infection in 3T3 cells overexpressing Unc119. Fyn was not significantly activated during infection in normal cells but was strongly activated in Unc119 overexpressing cells (Figure S2C). To explore if the Unc119 mediated regulation of bacterial infection is dependent upon SFKs, we used the SFK deficient SYF fibroblast cell line, which lacks Src, Yes and Fyn kinases. In the absence of all SFKs, Unc119 knockdown still doubled the bacterial infection in the SYF cell line (Figure S2D). This suggests that Unc119 inhibition of *Shigella* infection is not mediated by SFKs.

### Unc119 interacts with Abl/Arg tyrosine kinases and their substrate Crk

**Figure 3:**
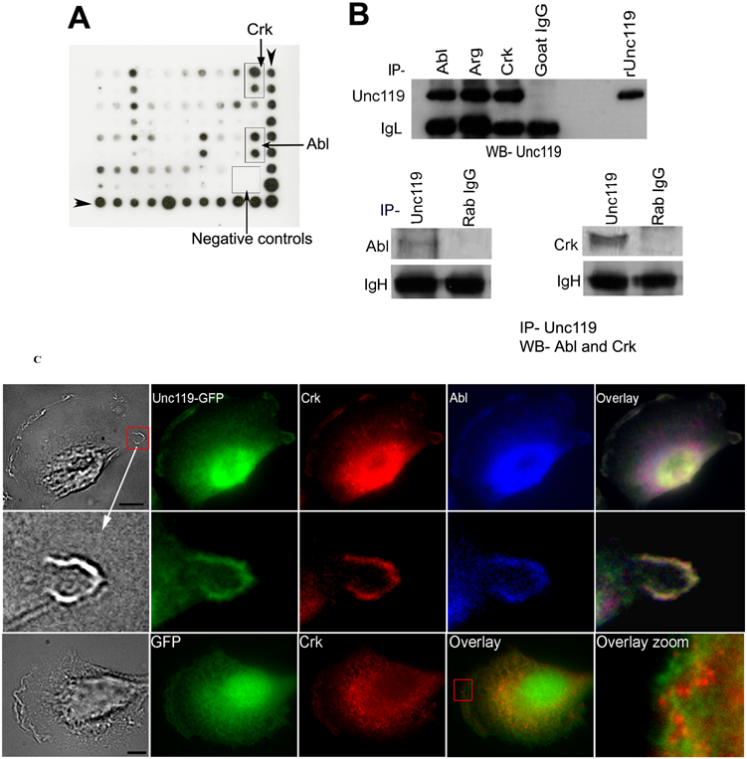
Unc119 interacts with Abl family kinases and Crk. (A) An array screen for Unc119-interacting SH3 domain proteins. Recombinant Unc119 was labeled with biotin and used to probe a protein array containing 38 SH3 domains from different proteins that were spotted undiluted and at 1∶5 dilution. Unc119 binding was detected by treating the membrane with streptavidin-linked HRP and its substrate (N = 2). Positive control spots are shown on the right last column and the bottom row (indicated by arrow heads). Arrows show the position of two select proteins (Crk and Abl). (B) Unc119 co-precipitates with Abl, Arg and Crk proteins. Abl, Arg and Crk proteins were immunoprecipitated with specific antibodies and co-precipitation of Unc119 was examined (Upper panel). IgL indicates protein loading. Conversely, Unc119 was immunoprecipitated with a rabbit anti-Unc119 antibody and co-precipitation of Abl and Crk with Unc119 was checked (lower panels, N = 3). IgH indicates protein loading. (C) Unc119 colocalizes with Abl and Crk. 3T3 cells were transfected with GFP-Unc119 or GFP vector, grown on cover glass over night. The cells were fixed and immunostained using antibodies against Crk (red) and Abl (blue). A bright field image shows the membrane folding. Middle panels are close-up images of a membrane fold indicated by the arrow. Lower panels shows control GFP-expressing cells (N = 3). Scale bar, 10 µm.

To identify proteins that interact with Unc119 and thus affect bacterial infection, we screened for Unc119 interactive SH3 domain-containing proteins using a TranSignal SH3 domain array. The array contained 38 SH3 domains of different cellular proteins. Unc119 interacted with the SH3 domain of a number of proteins. The order of binding to the top 4 SH3 proteins on the array was Abl>Crk>PSD95>spectrin (Figure 3A). Crk is a substrate for Abl and Arg kinases [25]. These molecules are involved in cell motility, membrane ruffles and bacterial infections making them the best candidates for further studies [18], [26]–[28]. To confirm the results of the array screen we immunoprecipitated Crk, Abl and Arg proteins from 3T3 cell lysates and checked for co-precipitation of Unc119. All three immunoprecipitates showed the presence of Unc119 (Figure 3B upper panel). Conversely, the immunoprecipitate of Unc119 showed the presence of Abl and Crk (Figure 3B, lower panel). Next we examined the association of Crk, Abl and Unc119 by immunostaining. We expressed Unc119 as a fusion protein of GFP and stained for Crk and Abl. Overexpression of Unc119-GFP resulted in altered cytoskeleton organization in the cell and reduced membrane ruffle formation (see below). In some cells where membrane ruffles were detectable, Unc119-GFP but not GFP localized to membrane ruffles and co-localized with Abl and Crk (Figure 3C). Similarly Arg kinase showed co-localization with Unc119 at the membrane ruffles (Figure S3). The co-precipitation and co-localization studies indicate that Unc119 forms complexes with Abl, Arg and Crk at membrane folds. This localization of Unc119 is important because membrane ruffles are active sites for bacterial engulfment [2].

### Unc119 regulates infection by inhibiting Abl/Arg kinase activity

**Figure 4:**
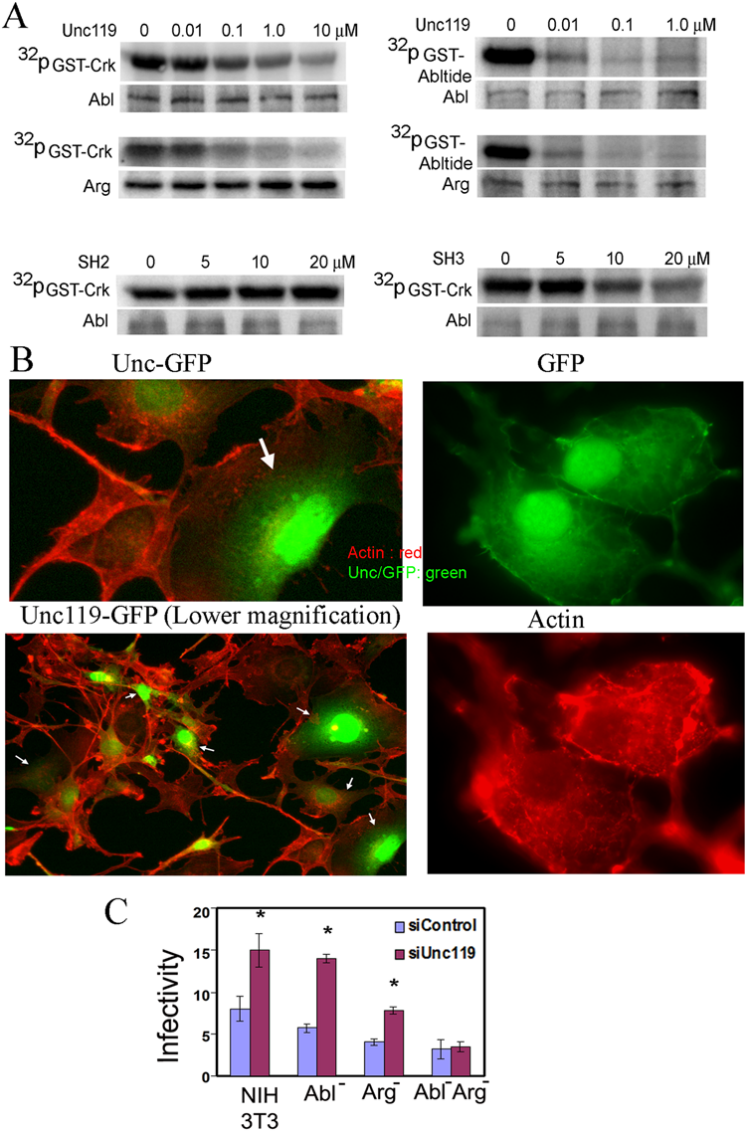
Unc119 blocks *Shigella* infection by inhibiting Abl family kinases. (A) Unc119 inhibits Abl and Arg kinase activity. Abl and Arg kinases were immunoprecipitated from the 3T3 cells and their kinase activity was measured by incorporation of ^32^P-γ-ATP into two different substrates- GST-Crk (left top two panels) and GST-Abltide (right top two panels) in presence of increasing concentrations of recombinant Unc119 (Top and middle panels) or Unc119-derived SH2 and SH3 motif peptides (bottom panels. The samples were then separated by SDS-PAGE, transferred to PVDF membrane, and exposed to storage phosphor screen. The phosphorylation was captured using the Typhoon Variable Mode Imager. The membranes were reprobed for the kinases to show equal loading (lower panels, N = 3). (B) Unc119 overexpression inhibits actin polymerization. 3T3 cells overexpressing Unc119-GFP (left two panels) or GFP (right panels) were stained for actin by phalloidin (left two panels and right bottom panel. The upper left panel is a higher magnification of three cells from the bottom left image (bottom right corner). Arrows indicate cells overexpressing Unc119-GFP (N = 3). (C) Unc119 knockdown has no effect on *Shigella* infection in the absence of Abl family kinase activity. 3T3, Abl^−/−^, Arg^−/−^ and Abl^−/−^Arg^−/−^ cells were transfected with siControl and siUnc119 RNA. After 48 h the cells were infected with *Shigella* and the infectivity was measured. The data represents mean±SD of 3 independent experiments performed in triplicates (*P<0.038).

As mentioned previously, Unc119 activates the Src family of tyrosine kinase. Since Abl and Arg kinases belong to a closely related tyrosine kinase family, we asked if Unc119 affected the kinase activity of Abl and Arg. We examined the effect of recombinant Unc119 on the enzymatic activity of Abl family kinases in an in vitro kinase assay using GST-Crk as a substrate. Unc119 inhibited the phosphorylation of GST-Crk in a dose-dependent manner (Figure 4A, left panels). The inhibitory effect was stronger on Arg than Abl kinase. Since Unc119 directly interacts with Crk through its SH3 domain, it is possible Unc119 sequesters Crk and thereby prevents its phosphorylation without affecting Abl kinase activity. To address this concern we used a different substrate that lacks the Crk SH3 domain. We used GST-Abltide, which is a fusion peptide containing the Abl phosphorylation motif (EAIYAAPFAKKK) with an N-terminal GST-tag. The inhibition of phosphorylation of GST-Abltide by Unc119 (Fig. 4A, right panels) was actually stronger than that of GST-Crk. The results suggest that the inhibitory effect of Unc119 on Abl kinases is direct. To demonstrate the region of Unc119 that interacts with the kinases and inhibits the enzymatic activity we used peptide sequences of the SH2-binding motif (TCEHIYDFPPLS) and SH3-binding motif (QGKQPIGPED) from Unc119 in the kinase assay. The presence of the SH2 binding motif had no effect on the kinase activity (Figure 4A, lower panels). On the other hand, the SH3-binding motif of Unc119 caused inhibition of GST-Crk phosphorylation indicating that an SH3-mediated interaction is responsible for inhibition of the enzymatic activity.

Abl and Arg kinases are known to induce actin polymerization [16], [21]. If the inhibitory action of Unc119 were important, it would affect actin polymerization. We examined the effect of Unc119 overexpression on actin polymerization. Overexpression of Unc119 as a GFP fusion protein showed reduced actin polymerization and reduced formation of dendrite-like cellular projections (Figure 4B). Overexpression of GFP alone had no effects. In agreement with these findings we observed flattening of cells that overexpressed Unc119 (Figure S4). This was associated with an apparent increase in the cell size in culture plates. There was a simultaneous reduction in the number of dendrite-like projections and membrane ruffles.

Abl kinases and their substrate Crk are known to play an important role in *Shigella* infection [16]. We asked whether the inhibition of bacterial uptake by Unc119 was mediated by Abl kinases. In Abl^−/−^ and Arg^−/−^ cells the bacterial infection increased in the absence of Unc119 to a degree that was similar to that observed in control cells (Figure 4C). However, in Abl^−/−^Arg^−/−^ cells the bacterial infection was unaffected by the absence of Unc119. The above results suggest that Unc119 inhibits *Shigella* infection by inhibiting Abl family kinases.

### Effect of Unc119 overexpression on *Shigella* infection and Abl family kinase activation

**Figure 5:**
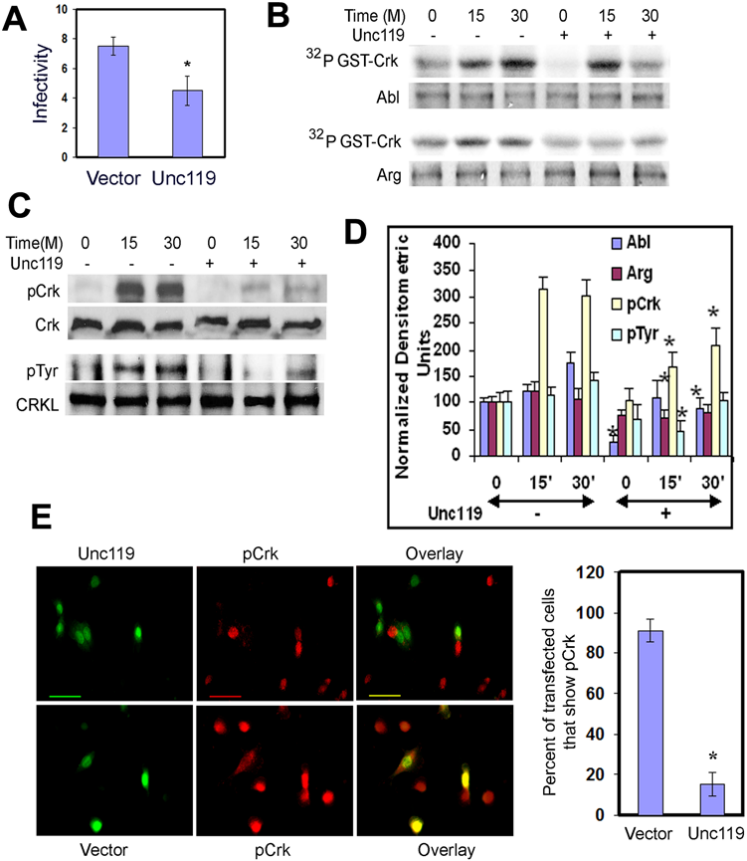
Overexpression of Unc119 inhibits *Shigella* infection, Abl/Arg activation and Crk phosphorylation. (A) 3T3 cells were infected with the bicistronic GFP-RV retrovirus (vector) or GFP-RV-Unc119 retrovirus (Unc119). Cells were sorted for GFP expression and infected with *Shigella*. The results represent mean±SD of 3 independent experiments performed in triplicates (*P = 0.025). (B) Reduced Abl and Arg kinase activity ex vivo in Unc119 overexpressing cells. 3T3 cells expressing GFP-RV-Unc119 and GFP-RV were infected with *Shigella* and the cell lysates were collected at the noted time points. Abl and Arg kinases were immunoprecipitated and assayed for kinase activity using recombinant GST-Crk as a substrate. (C) Inhibition of Crk and CRKL phosphorylation by Unc119. Crk and CrkL were activated by *Shigella* and PDGF (20 ng/ml) respectively. Their tyrosine phosphorylation was examined after immunoprecipitation by western blotting using an anti-pCrk (Y^221^) or anti-phosphotyrosine (4G10) antibody. Equal loading was checked after reprobing the membranes with an anti-CrkII (Crk) and anti-CrkL antibody. (D) Densitometric analysis of 3 independent experiments from Figures B & C is shown. *P<0.05 compared to the same time point in the absence (-) of Unc119. (F) Reduced Crk phosphorylation in Unc119 overexpressing cells. Cells plated on a glass cover-slip were transiently transfected with GFP-RV-Unc119 and GFP-RV followed by infection with *Shigella*. The cells were immunostained for pCrk (red). The green cells (GFP+) indicate the expression of GFP and Unc119 on the upper panel or GFP alone on the lower panel. Scale bar, 20 µm. The right bar graph shows the number of GFP-RV-Unc119 (Unc119) and GFP-RV (vector) expressing cells that also show Crk phosphorylation. The results represent mean±SD of three independent experiments (*P<0.001).

Next, we examined the effect of overexpression of Unc119 on bacterial uptake. Unc119 overexpression by a bicistronic GFP-RV-Unc119 retrovirus reduced bacterial uptake by 40% (Figure 5A). Overexpression of proteins can be toxic to many cell types. To ensure that the reduced bacterial uptake was not due to the loss of cell viability, we measured LDH release from Unc119 overexpressing cells, which was similar to that from control vector-transfected cells (data not shown). Next, we studied the activation of Abl kinases and Crk phosphorylation during bacterial infection in cells overexpressing Unc119. Abl and Arg kinases were immunoprecipitated and the kinase assay was performed using GST-Crk as a substrate. In Unc119 overexpressing cells the Abl and to a lower extent, the Arg kinase activity was low compared to the cells expressing the vector alone (Figure 5B & D). This data suggest that in the presence of Unc119 Abl and Arg kinases are not fully activated by bacterial infection. The decreased Abl family kinase activation should result in decreased phosphorylation of their physiological substrates Crk and CrkL [29]. Indeed, tyrosine phosphorylation of Crk (Y^221^) by *Shigella* infection and to a lower extent, phosphorylation of CrkL by PDGF was decreased in Unc119 overexpressing cells (Figure 5C & D) at 15 and 30 min after bacterial infection. The decrease in Crk phosphorylation was also examined by immunofluorescent staining of the cells following *Shigella* infection. Absent or reduced Crk Y^221^ phosphorylation was observed in 85% cells overexpressing Unc119 as indicated by GFP co-expression (Figure 5E). This contrasted with Crk Y^221^ phosphorylation during infection in cells overexpressing GFP alone.

### Cell-permeable Unc119 inhibits *Shigella* infection in a mouse model

We generated cell-permeable Unc119 and Unc119 (P59A, P62A), an SH3 motif mutant, by constructing fusion proteins with the HIV TAT sequence [30] (Figure S5A). The TAT-GFP fusion protein was used as a negative control. To confirm that TAT mediates protein transduction into cells we incubated 3T3 cells with the fusion proteins. Flow cytometric and fluorescence microscopic studies confirmed the uptake of the TAT-GFP fusion protein (Figure S5B). Cells internalized TAT-Unc119 efficiently compared to Unc119 without TAT (Figure S5C). The uptake of the TAT-Unc119 protein reached the peak in about 1 h and showed a linear dose-dependent relationship (Figure S5D). To study bacterial uptake cells were incubated with TAT-Unc119, TAT-Unc119 mutant and TAT-GFP proteins at 1 µM concentration for 1 h and then infected with *Shigella*. The *Shigella* uptake was reduced by 66% in TAT-Unc119 transduced cells compared to TAT-GFP transduced cells (Figure S5E). The infection was only slightly but non-significantly reduced, when the cells were transduced with the TAT-Unc119 mutant protein indicating that the inhibition needs an intact SH3 binding motif.

**Figure 6:**
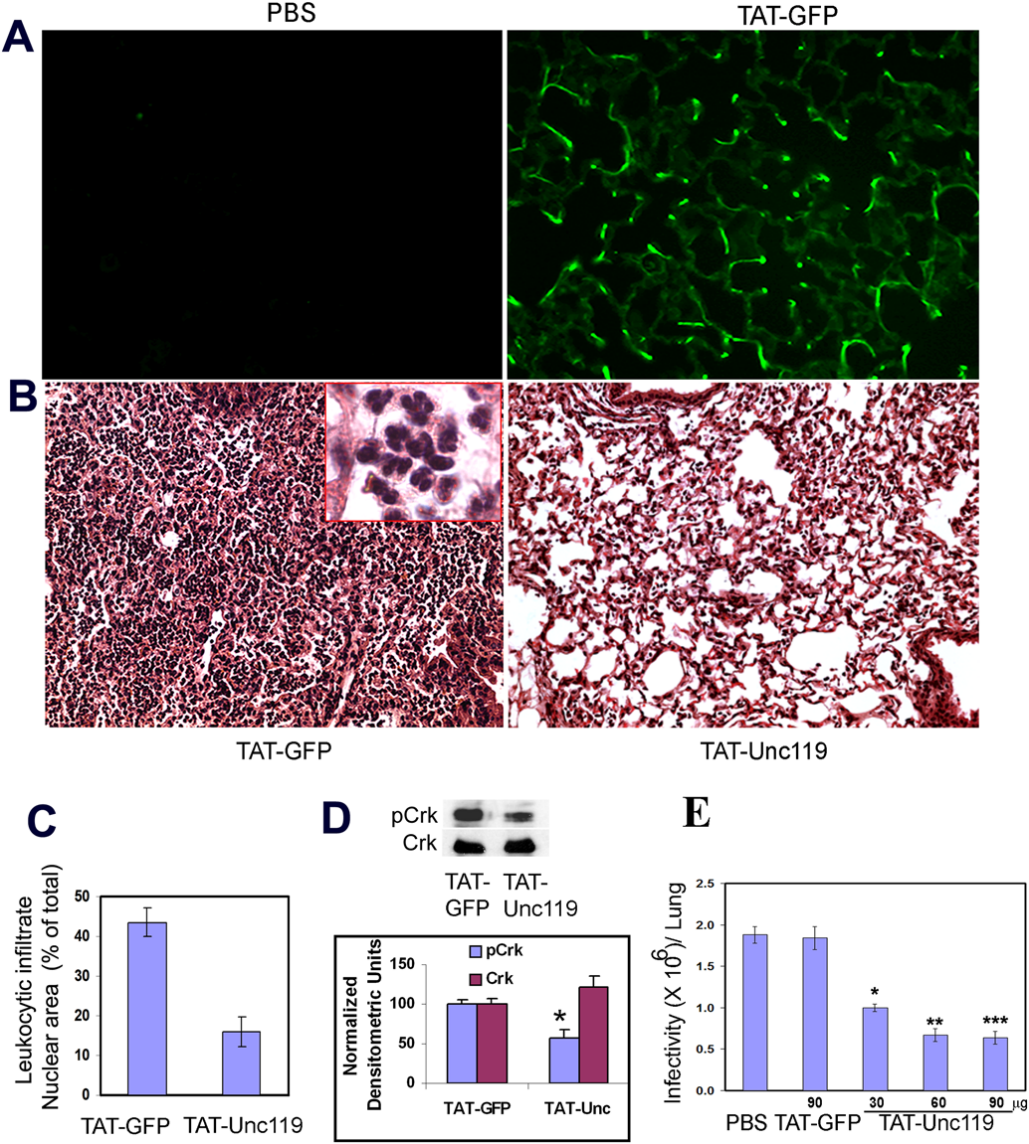
Unc119 inhibits mouse pulmonary shigellosis. (A) Transduction of TAT-GFP fusion protein in vivo. Mice were transduced intranasally with PBS and TAT-GFP (30 µg/mouse) and the uptake of GFP by airway cells is shown (N = 3 for each group). (B) Inhibition of *Shigella*-induced lung inflammation by TAT-Unc119. Mice were transduced with TAT-GFP (left panel) and TAT-Unc119 (right panel) (30 µg/mouse) and then infected as described above. Lung sections were obtained 24 h later and stained with H & E. The inset shows a higher magnification of the leukocytic infiltrate. Images are representative of 3 mice per group. (C) Decrease in *Shigella*-induced leukocytic infiltration after TAT-Unc119 treatment. The alveolar, peribronchial and perivascular areas with leukocytic infiltrates were measured and expressed as percent of the total area. The data represents average of 20 random fields from 3 mice per group. (D) Inhibition of Crk phosphorylation by TAT-Unc119 in vivo. Lung lysates from TAT-GFP and TAT-Unc119 treated mice were used to immunoblot for pCrk followed by reprobing for Crk. The bottom panel shows densitomtric analysis of the immunoblot from 3 experiments. P<0.04 for pCrk, TAT-Unc vs TAT-GFP. (E) Inhibition of pulmonary *Shigella* infection by TAT-Unc119. Mice were treated intranasally with the indicated concentration of PBS, TAT-GFP or TAT-Unc119 and then infected with *Shigella* (1×10^6^/ mice) 1 h later. The infectivity was measured by plating the lung homogenate 6 h after infection. The results represent mean±SD of bacterial counts from 6 mice per group (*P = 0.007, **P = 0.0016, ***P = 0.0022).

Next, we studied the effect of this cell-permeable form of Unc119 in a mouse model of pulmonary shigellosis [31], [32]. Mice were treated intranasally with PBS, TAT-GFP or TAT-Unc119 followed by infection with *Shigella*. The uptake of TAT-GFP by lung cells was confirmed by fluorescence microscopy (Figure 6A). TAT-GFP transduced lungs showed severe inflammation at 24 h after *Shigella* infection (Figure 6B, left panel), which was similar to that seen in the PBS-treated mice (not shown). The inflammation was characterized by the presence of neutrophils and to a lower extent, mononuclear cells in the alveolar space as well as in peribronchial and perivascular areas. Pretreatment with TAT-Unc119 resulted in decreased neutrophilic inflammation (Figure 6B), right panel. The leukocyte infiltration was 3 fold less in the lungs treated with TAT-Unc119 (Figure 6C). The treatment of mice with TAT-Unc119 was associated with reduced Crk phosphorylation (Figure 6D, upper and lower panels). The infectivity in PBS and TAT-GFP treated lungs was similar indicating that TAT-GFP had no effect on bacterial infection. The lung infectivity (number of *Shigella* per lung) in TAT-Unc119 pre-treated lungs was decreased in a dose-dependent manner (Figure 6E). At the 30 µg dose per mouse the lung infectivity was half of the control and at the 90 µg dose the lung infectivity was only one third.

### Unc119 is an inducible protein

**Figure 7:**
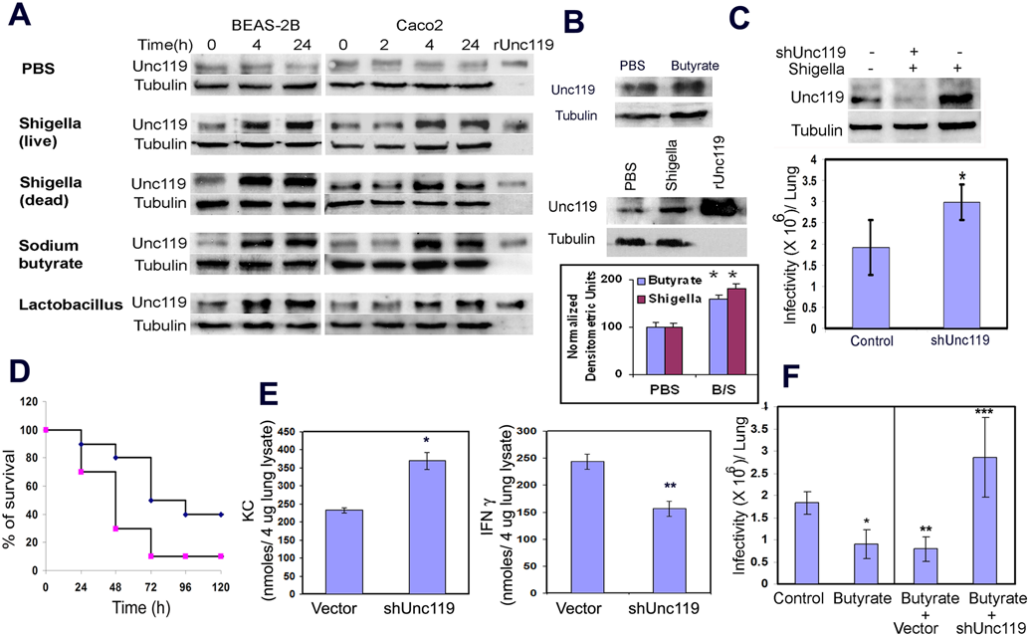
Unc119 is essential for protection against *Shigella* infection. (A) Induction of Unc119 expression. BEAS 2B (left panel) and Caco2 (right panel) cells were incubated with PBS, live and dead *Shigella*, sodium butyrate and probacteria Lactobacillus acidophilus for the indicated time period and western blotted for Unc119. rUnc119 indicates the position of recombinant Unc119 in the gel. The membranes were reprobed with an anti-tubulin antibody to check for equal protein loading (N = 3). (B) Unc119 induction in mouse lungs. Mice were pretreated with 20 µl of sodium butyrate (1 M, top panel) in PBS, *Shigella* (10^6^/mouse, middle panel) or PBS alone and the expression of Unc119 was measured after 24 h. The bottom bar graph shows densitometric analysis of Unc119 induction by butyrate (B) and *Shigella* (S) from 3 experiments. *B/S vs. PBS p<0.02. (C) Augmentation of pulmonary shigellosis in Unc119-deficient lungs. Mice were transfected intranasally twice, 24 hr apart, with a shUnc119 vector and the control vector in ExGen 500. Next day mice were infected with *Shigella* (1×10^6^/ mice). The expression of Unc119 (upper panel) and infectivity (lower panel) was measured 6 h later. The results represent mean±SD of bacterial counts from 4 mice per group (*P = 0.039). (D) Increased lethality of *Shigella*-infected Unc119-deficient mice. Control (blue diamonds) and shUnc119 (pink squares)-treated mice were infected with *Shigella* (1×10^7^ /mice) and survival was measured for up to 120 h. Ten mice were used for each group. The difference in survival between the groups was significant (p = 0.04, Chi-square test). (E) Cytokine expression during the *Shigella* infection in Unc119-deficient lungs. The cytokine levels of KC and IFN-γ were measured in the lung lysates 6 h after infection. (F) Inhibition of pulmonary *Shigella* infection by sodium butyrate requires Unc119. Mice were treated with 20 µl of sodium butyrate (1 M) in PBS or PBS (control) alone (left two bars). They were then infected with *Shigella* 24 h later. Infectivity of the lung tissue was measured 6 h after the infection. In another set of experiments mice were first transfected with shUnc119 or the control vector. After 24 h they were treated with 20 µl of sodium butyrate (1 M) in PBS (right two panels). The mice were infected with *Shigella* 24 h later and infectivity measured. The results represent mean±SD of bacterial counts from 6 mice (*P = 0.0004, ** P = 0.00005, *** P = 0.019).

We wondered whether Unc119 was an inducible protein and regulated by factors that secondarily affected infection. To this goal we studied the effect of *Shigella* (live and dead), LPS and probacteria (Lactobacillus acidophilus) on Unc119 expression. We also studied sodium butyrate, a bacterial product in the gut, which affects cell function by inhibiting histone deacetylase. Sodium butyrate has recently been shown to inhibit *Shigella* infection in rabbits [33]. In vitro *Shigella* (live and dead), probacteria and butyrate induced Unc119 expression in lung (BEAS-2B) and colonic (Caco2) epithelial cells (Figure 7A). LPS, a major component of the bacterial cell wall, also induced Unc119 in lung epithelial cells (Fig. S6). Next we examined Unc119 expression in the mouse lung. Treatment with butyrate upregulated the expression of Unc119 in the lungs (Figure 7B, top and bottom panels). Like butyrate *Shigella* infection also increased Unc119 expression in the lungs in vivo (Figure 7B, middle and bottom panels). We speculate that the upregulation of Unc119 by *Shigella* is a homeostatic response that is aimed at preventing further infection.

### Unc119 deficiency augments pulmonary shigellosis

We wanted to examine the effect of Unc119 knockdown on *Shigella* infection in vivo. To this goal we generated an shRNA construct for Unc119 in a Banshee vector that knocked down Unc119 expression by 70% in fibroblasts (Fig. S7). Intranasal treatment of mice with the shUnc119 construct induced a 71% reduction in Unc119 expression in infected mouse lungs (Fig. 7C, upper panel). This reduction in Unc119 expression was associated with a statistically significant (*P = 0.039) increase in *Shigella* infectivity (Figure 7C, lower panel). *Shigella* count in the lungs was increased by 51% in shUnc119-treated mice as compared to control vector-treated mice. In Unc119 knockdown mice *Shigella* infection is more lethal and caused increased animal death (Figure 7D).

Various cytokines influence *Shigella* infection in mouse. IL8 and IFN-γ are known to play a role in *Shigella* infection [34], [35]. The murine chemokine KC is a homologue of human IL8. We checked level of KC and IFN-γ in control and shUnc119 knock down lungs during the infection. Unc119 knockdown almost doubled the KC levels in the lungs (Figure 7E). On the other hand IFN-γ levels went down in shUnc119-treated lungs.

Sodium butyrate has previously been shown to reduce *Shigella* infectivity in rabbits [33]. To examine whether Unc119 upregulation is one of the mechanisms by which sodium butyrate decreases the bacterial infection we treated Unc119 knockdown mice with butyrate and studied *Shigella* infection. Treatment with butyrate decreased *Shigella* infection in the mouse lung (Figure 7F, left panel). However, in the absence of Unc119 butyrate was ineffective in decreasing the bacterial infection (Figure 7F, right panel) indicating that Unc119 plays an essential role in mediating the protective effect of butyrate.

## Discussion

*Shigella* and other pathogens engage many cellular proteins such as Src and Abl family kinases, Crk and cortactin to facilitate their entry into the cell [5], [9]. It is unknown whether this process leads to the engagement of cellular inhibitory mechanisms to counter the pathogen entry. In this study we demonstrate that Unc119 functions to regulate *Shigella* infection. The inhibitory effect of Unc119 on *Shigella* infection is similar regardless of the cell line used suggesting that it represents a general inhibitory mechanism. Unc119 also blocks the uptake of BCG by the THP1 cells suggesting that the effect is not specific for *Shigella*.

Through an SH3 domain array screen we identify Abl family kinases and their target Crk as novel binding partners and effectors of Unc119. The interaction of Unc119 with Abl family kinases and Crk is important for a number of reasons. Both Abl and Arg kinases regulate actin cytoskeleton [36], [37]. Arg kinase interacts with both actin and tubulin and promotes their cross-linking [18]. We show that Unc119 co-localizes with both Abl and Arg kinases and their substrate Crk. Unc119 also co-localizes with *Shigella*. *Shigella* engages CD44 as one of the host molecules to enter mammalian cells [5], [6]. We have shown that CD44 physically interacts with Unc119. We speculate that CD44 recruits Unc119 to the site of *Shigella* invasion.

One of the most important findings of this work is the inhibition of Abl family kinases by Unc119. It directly inhibits Crk phosphorylation by Abl kinases. Crk phosphorylation by Abl/Arg kinases results in activation of Cdc42 that leads to actin rearrangement and internalization of bacteria [16]. Cells deficient in Abl kinases or expressing non-phosphorylatable mutants of Crk are resistant to *Shigella* infection. Recent studies have shown that Abl kinases play an important role in invasion of cells by Chlmydia trchomatis and Coxsackievirus [38], [39]. They are also essential in poxvirus dissemination [40]. In both studies the inhibition of Abl family kinases by imatinib blocked the infection. We believe that the inhibition of Abl kinases by Unc119 directly contributes to its inhibitory effect on *Shigella* infection. Unc119 knockdown had no effect on infection in Abl^−/−^Arg^−/−^ cells. We examined the mechanism of inhibition of Abl/Arg kinases by Unc119. Our results suggest that the SH3 binding motif of Unc119 but not the SH2 binding motif is essential and sufficient for inhibition of Abl and Arg kinases. A number of proteins including F-actin, Abi1, Abi2, AAP1, PAG and RB1 and phosphatidyl inositol 4,5 biphosphate interact with Abl kinases and inhibit their kinase activity [41]–[48]. Except for F-actin and the phospholipid the other proteins exert their inhibitory effect through the interaction with the SH3 domain. This is consistent with the finding that deletion or mutation of the SH3 binding motif of Unc119 restores activation of Abl kinases.

To demonstrate that Unc119 plays a role in *Shigella* pathogenesis in vivo we performed a proof-of-concept experiment in a mouse model of shigellosis [31], [49]. Mouse colonic epithelium is resistant to *Shigella* infection. As an alternative investigators have developed a mouse model of pulmonary shigellosis. In this model transduction of Unc119 inhibits *Shigella* infection and neutrophilic inflammation. Conversely, Unc119 knockdown increases Shigellla infection and mortality. It should be emphasized that *Shigella* is specifically adapted to human colon. Thus, the results of our experiments with cell lines and pulmonary shigellosis must be interpreted with reservations until the relevance of Unc119 is confirmed in humans.

In absence of Unc119, the level of IFN-γ decreases. IFN-γ has been shown to be essential for innate resistance to *Shigella* infection in mouse lungs and in its absence the mouse become more susceptible to infection and manifest increased mortality [34]. Mouse KC is a functional homologue of human IL8. The level of KC increases with bacterial infection [33], which is likely a defense mechanism and increases the recruitment of neutrophils. The increase in the level of KC in Unc119-deficient lungs could contribute to the worsening pathophysiology and increased mortality. The exact mechanism of the altered cytokine level in Unc119-deficient lungs is not known. Unc119 plays an important role in mediating intracellular signals for cytokine (e.g. TGF-β, IL-5) receptors and T cell antigen receptors [19]–[21]. During *Shigella* infection the activation of some of these receptors may be impaired in the absence of Unc119, which could result in altered cytokine production. It should be pointed out that the changes in the cytokine level could be secondarily due to the increased inflammation.

Unc119 is an inducible protein and *Shigella* infection increases the expression of Unc119 in the mouse lungs, human lung and colonic epithelial cells. Lactobacillus, a normal gut flora, also upregulates Unc119. Intestinal infection with pathogenic bacteria frequently occurs when the normal gut flora is destroyed (e.g. after an antibiotic treatment). We speculate that the loss of Lactobacillus reduces Unc119 expression and thereby, increases susceptibility to pathogens. Butyrate is a fermentation product of the normal gut flora and provides protection against *Shigella* [32]. Butyrate was shown to induce the antimicrobial peptide LL-37 in rabbit colonic epithelium [32]. We show that butyrate upregulates Unc119. We speculate that the normal gut flora supports the expression of Unc119 in epithelial cells directly and also indirectly through the generation of butyrate. A transient loss of or reduction in Unc119 expression along with other innate host factors allows *Shigella* invasion of colonic epithelial cells. *Shigella* infection, although serious, is nonetheless a self-limited illness in most healthy subjects. During *Shigella* infection Unc119 is rapidly induced to counter the persistence of the bacterial invasion, which results in spontaneous recovery. Sodium butyrate has recently been promoted as a therapeutic agent for *Shigella* infection [32]. We show that this therapeutic benefit of butyrate is lost in the absence of Unc119. We propose the induction of Unc119 as a novel approach to boosting host defense and fighting infection.

**Figure 8:**
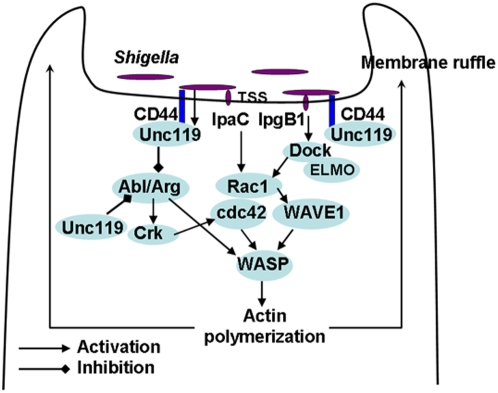
A schematic presentation of the action of select *Shigella*-derived effector proteins on the host cell and the mechanism of inhibition of *Shigella* invasion by Unc119.

In summary, *Shigella* infection elicits two opposing signaling events (Figure 8). Through the activation of tyrosine kinases and Rho/Rac family of GTPases it induces actin polymerization and cytoskeletal reorganization, which promotes its own uptake. *Shigella* also recruits Unc119 through CD44, which inhibits Abl/Arg tyrosine kinases and Crk phosphorylation. This leads to an inhibition of bacterial uptake. *Shigella* stimulates Unc119 synthesis, which further boosts this inhibitory pathway. We speculate that this Unc119-mediated inhibition represents a non-immunologic recovery mechanism in certain infections.

## Methods

### Cell cultures and media

Mouse Embryonic fibroblasts (NIH 3T3, CRL-1658), Src, Yes, Fyn knock out fibroblasts (SYF, CRL-2459), epithelial (Caco-2, HTB-37), lung epithelial (BEAS-2B, CRL-9609), monocytes (THP-1, TIB 202) cell lines were obtained from American Type Culture Collection (ATCC). The fibroblast, Caco-2 and monocyte cell lines were maintained in DMEM, MEM, RPMI1640 (Mediatech, Herndon, VA) supplemented with 10% fetal calf serum (Cellgro, Herndon, VA) respectively. For serum starvation of Caco2 cells, the cultures were grown on MEM supplemented with ITS (insulin, transferrin and selenium). BEAS-2B cells were maintained on BEBM with supplements (Lonza, Walkersville, MD). THP-1 cells were differentiated into adherent, macrophages by the addition of phorbol myristate acetate (PMA, 20 ng/ml) 24 h before infection studies. HeLa cells were a kind gift of Dr. Erwin W. Gelfand, National Jewish Medical and Research Center, Denver, CO. HeLa cells were grown in DMEM. Abl and Arg knockout fibroblasts were kind gift from Dr. Steven Goff of Columbia University, New York, NY and Dr. A. Koleske of Yale University School of Medicine, New Haven, CT. Medium was changed every 3 days. Cells were plated on 35- or 100-mm dishes and incubated in 5% CO_2_ air at 37°C. For immunofluorescence studies, cells were plated on glass cover slips and placed in 6-well plates.

### Plasmids and constructs

Mouse Unc119 cDNA was cloned into the bicistronic retroviral vector GFP-RV as described previously [20]. For generation of Unc119 overexpressing fibroblasts, GFP-RV-Unc119 retrovirus was produced by transfecting the Phoenix Ampho packaging cell line using TransFectin (Bio-Rad laboratories, Hercules, CA) as per manufacturer's protocol. The supernatants were collected 48 h after transfection and sterilized with 0.45-µm filters. Target cells were suspended in viral supernatants mixed 1∶1 with the growth media (DMEM+10% FCS) and 8 µg/ml polybrene and then centrifuged in plates at 1200 g for 90 min at 32°C. The cells expressing Unc119 were selected at 48 hours by sorting for the GFP-expressing cells. For Unc119-GFP fusion protein the Unc119 cDNA was cloned into the pLEGFP vector (Clontech, Mountain View, CA). The plasmid was transfected into 3T3 cells using lipofectamine 2000 (Invitrogen, Carlsbad, CA) or the Amaxa Nucleofector kit (Amaxa, Inc. MD). Recombinant Unc119 was expressed as a GST fusion protein in the pGEX2T vector by inserting the Unc119 sequence in-frame at EcoRI and SalI restriction sites. For construction of the TAT fusion protein, the TAT sequence (MRKKRRQRRR) followed by Xba1 site was inserted into the pGEX2T-Unc119 vector by PCR mutagenesis to produce pGEX2T-TAT-Unc119. For SH3 domain mutation, the amino acids P^59^ and P^62^ were changed to alanine by PCR mutagenesis. The EGFP sequence from the pLEGFP vector (Clontech) was amplified and inserted in-frame into the pGEX2T-TAT-Unc119 vector at Xba1 and Xho1 sites replacing Unc119 sequence to construct the pGEX2T-TAT-GFP vector. The sequence and the insertion sites were confirmed by sequencing. The recombinant proteins were expressed and purified using glutathione agarose beads. The GST sequence was cleaved by thrombin. The TAT-Unc119 and TAT-Unc119 mutant proteins were passed through a Sephadex G-50 column. The peak fractions were pooled and the purity of the proteins was checked by polyacrylamide gel electrophoresis followed by Coomassie blue staining.

### Bacterial culture and infection protocols

The *Shigella* flexneri 2457T (serotype 2a, ATCC 700930), S. flexneri CVD1203 (ΔaroA ΔvirG, ATCC 55556) and Lactobacillus acidophilus (ATCC 53545) were obtained from the American Type Culture Collection. Mycobacterium bovis BCG is a kind gift from Dr. Kevin Kisich, National Jewish Medical and Research Center, Denver, CO. *Shigella* wild type bacterial colonies were picked from the Congo Red agar plates and then grown on tryptic soy broth (TSB) agar plates and in liquid cultures. Lactobacillus was cultured on Man-Rogosa-Sharpe (MRS) broth. Mycobacterium was grown in Middlebrook 7H9 medium. For infection, overnight cultures of *Shigella* were diluted 1∶100 and allowed to grow to logarithmic phase (OD_600_ = 0.5). The gentamicin protection assay was performed as described previously [50]. Briefly, the bacteria were grown to mid-logarithmic phase, then washed and resuspended in DMEM containing 10% FBS and 50 mM HEPES pH 7.3. The bacteria were added to cell cultures at a multiplicity of infection of 100. Spin-infection was carried out centrifuging the culture plates at 700 g for 10 min and then incubating at 37°C for 2 h. The cells were washed twice with DMEM and then incubated for 2 h with the medium containing gentamicin (50 µg/ml). The number of cells per well was counted from duplicate wells by trypsinizing the cells. The cells were lysed with 1% Triton X-100 and serially diluted lysates were plated on TSB agar plates. Next day the number of colony forming units (CFU) was counted from the plates and the infectivity was calculated as number of CFU per 10 cells. For production of *Shigella* expressing the red fluorescent protein the bacteria were transfected with the DsRed vector (Clontech) and selected on ampicillin plates. For quantitation of infectivity by flow cytometry the THP-1 cells were tryspinized after 1 h of infection and then fixed in paraformaldehyde for 1 h. The cells were permeabilized with 0.05% saponin in PBS. The internalized bacteria were stained using a rabbit anti-*Shigella* antibody. For Mycobacterium uptake, the bacteria were pre-labeled and then incubated with THP-1 cells (**Figure. S1B**).

### Animal experiments

All procedures on mice were approved by the National Jewish Medical and Research Center Institutional Animal Care and Use Committees and performed according to committee guidelines. All experiments were performed with 8 weeks old C57/B6 female mice. The mice lungs were transduced with the recombinant proteins intranasally in 30 µl PBS/mouse. After one hour the mice were infected intranasally with bacteria (1×10^6^/mouse). The mice were sacrificed 6 h later and the lungs were homogenized. Serial dilutions of the lysates were plated for bacterial colony forming units to determine the bacterial burden in lungs. For histological evaluation lungs were perfused with 10% formalin and embedded in paraffin. Hematoxylin and eosin (H&E) staining of the lung sections was done at the National Jewish Medical and Research Center Histology Core laboratory. For Unc119 knockdown the lungs were transfected intranasally with a shUnc119 and a control vector using ExGen 500 (Fermentas, Glen Burnie, MD) as per manufacturer's instruction. Each mouse was transfected twice, 24 h apart, with 25 µg of the vector in 50 µl of 5% glucose. The lungs were infected intranasally with *Shigella* (1×10^7^/mouse) after 24 h. For butyrate experiments mice were pretreated intranasally with a 20 µl aliquot of 1 M sodium butyrate in PBS and infected with *Shigella* (1×10^6^/mouse) 24 h later.

### Antibodies and fluorescence reagents

A rabbit polyclonal anti-Unc119 antibody was produced as described in Cen et al [19]. For affinity purification rabbit anti-Unc119 serum was passed through an Unc119 affinity column. Bound antibodies were eluted at low pH and stored in 10% glycerol. Antibodies against Fyn, Abl, Arg, CrkL, CrkII, CD44, integrin α5, tubulin (Santa Cruz Biotechnology, Santa Cruz, CA), Mouse CrkII (BD Transduction Laboratories, San Diego, CA), Phospho-Crk Y^221^ (Cell Signaling Technology, Danvers, MA), Phosphotyrosine (4G10) (Upstate Biotechnology, Charlottesville, VA) and *Shigella* (Biodesign International, Saco, ME) were used for immunoprecipitation, staining and western blotting. Neutralizing antibodies against integrin α5 and CD44 are from Chemicon International (Temecula, CA) and Endogen (Rockford, IL) respectively. Labeled secondary antibodies (Alexa 488, Alexa 594 and Alexa 350) were from Molecular probes (Invitrogen, Carlsbad, CA).

### Immunoprecipitation and Western blotting

For immunoprecipitation cells were lysed in a lysis buffer containing 50 mM Tris-HCl (pH 7.4), 75 mM NaCl, 1 mM EDTA, 1 mM NaF, 1 mM Na_3_VO_4_, 0.5% Nonidet P-40, 1 µg/ml each of the protease inhibitors aprotinin, leupeptin, pepstatin and 1 mM PMSF at a density of 2×10^6^ cell/100 µl of the lysis buffer. The cell lysate was incubated on ice for 30 min and detergent-insoluble materials were removed by centrifugation at 4°C at 13,000×g. The lysates were precleared with 20 µl of protein A/G agarose/100 µl of lysate for 1 h. An appropriate antibody (2 µg) was added to the lysate and incubated at 4°C rotating for 1 h or overnight followed by addition of 20 µl of protein A/G agarose. The incubation was continued for 2 h or overnight. The mixture was centrifuged at 12,000×g for 5 min and the pellet was washed three times with 1 ml of lysis buffer or three times with the kinase buffer in case of a kinase assay. The samples were separated by SDS-PAGE and transferred to polyvinylidene difluoride (PVDF) membranes for immunoblotting. The membranes were incubated in 5% BSA in TBST buffer for 1 h followed by incubation in the primary antibody at a concentration of 0.2 µg/ml in 5% BSA/TBST buffer. The membranes were then washed three times in the TBST buffer for 10 min each and incubated with a HRP-conjugated secondary antibody solution. After additional washing, the membranes were developed with the ECL (or ECL Plus) substrate. To strip and reprobe the membranes were incubated in the stripping buffer (100 mM 2-ME, 2% SDS, 62.5 mM Tris-HCl (pH 6.7)) at 55°C for 30 min, washed, blocked and immunoblotted with an appropriate antibody as described above. Densitometric analyses of select western-blotted protein bands were performed with the software “ImageJ” (http://rsb.info.nih.gov/ij/).

### Fluorescence Microscopy

Fluorescence images were obtained using Nikon 2000 Epifluorescence microscope fitted with a Coolsnap CCD camera. The camera and appropriate excitation and emission filter wheels were controlled by the Metamorph software (Molecular Devices, Sunnyvale, CA). Cells were grown on cover slips and fixed for 20 min by incubating in 4% phosphate-buffered paraformaldehyde. Cells were permeabilized for 30 min by incubating with 0.05% saponin in PBS. Samples were blocked for 30 min in a blocking solution containing 10% goat serum and 0.05% saponin in PBS, followed by 1 h incubation with the primary antibody. After 3 washes with PBS cells were incubated with an appropriately labeled secondary antibody for 1 h. Cover slips were then washed three times, mounted in the gel-mount mounting medium (Biomedia, Foster City, CA), and sealed on a slide with clear nail polish. For live infection cells expressing GFP-Unc119 were grown on delta T dishes over night and then infected with freshly cultured *Shigella* expressing DsRed. The dish was maintained at 37°C using the Delta T dish controller. The time-lapse images were collected every 30 sec, for 15 min for both green and red wavelengths using the Metamorph software.

### Generation of Unc119-deficient cells

Small interfering RNA (siRNA) for Unc119 and control non-targeting siRNA (Dharmacon, Denver, CO) were introduced into 3T3 cells in 6-well plates using the transfecting reagent Lipofectamine 2000 (Invitrogen ) according to the manufacturer's instructions. For shRNA the Unc119 sequence was ligated into the Banshee vector as described previously [51]. One of the clones with sequence GAGAGGCACUACUUUCGAAUU in the loop was selected for its better performance and used for further studies. Immunoblotting confirmed Unc119 knock down in the cells.

### Cytotoxicity measurement

LDH released from damaged cells was measured to estimate cytotoxicity using the LDH-Cytotoxicity Assay Kit (BioVision, Mountain View, CA). LDH release into the medium after 2 h of *Shigella* infection was calculated as a percent of total LDH, measured after lysis of cells by 1% triton X-100.

### Cytokine Measurement

Mouse CXC chemokine KC and IFN-γ were measured in the lung lysates obtained 6 h after bacterial infection using ELISA kits from the R&D systems, Minneapolis, MN and BD Biosciences, San Diego, CA, respectively.

### In vitro kinase assays

The kinases were immunoprecipitated with the respective antibodies from the cell lysate (2×10^6^ cells/assay) and then washed extensively. The immunoprecipitates were used in a kinase assay either in the absence (autophosphorylation assay) or presence of a substrate (e.g. GST-Crk or GST-Abltide). The assay was initiated by suspending the immunoprecipitate in a kinase buffer containing 20 mM Tris (pH 7.4), 2 mM MgCl_2_, 0.5 µM cold ATP, and 2 µCi 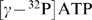 (Amersham Biosciences, Piscataway, NJ) and incubating for 15 min. The kinase reaction was stopped by addition of 4× Laemmli's buffer. The samples were separated on a 12% polyacrylamide gel (SDS-PAGE), transferred to PVDF membrane, and autoradiographed. For quantitation of the kinase activity the membranes were exposed to a storage phosphor screen and the activity was measured using the Typhoon Variable Mode Imager. The quantity was estimated by ImageQuant software (Amersham Biosciences).

### Statistical analysis

Two-sample t-test and Chi square test were used to evaluate statistical significance.

## Supporting Information

Figure S1 Unc119 knockdown increases the infectivity. A) Infectivity of S. flexneri CVD1203 ( aroA virG) mutant in 3T3 cells. The 3T3 cells were allowed to grow for 48 h followed by infection with Shigella flexneri mutant at a 1∶100 ratio and incubated for 2 h. The cells were washed and the extracellular bacteria were killed with gentamicin. The cells were lysed with 1% Triton X-100 and serial dilutions were plated for colony formation. The bacterial colony forming units were counted and the infectivity was expressed as the number of colony forming units per 10 cells. The results represent mean±SD of six independent experiments. *P = 0.006. (B) Uptake of BCG. To evaluate uptake of Mycobacterium bovis (BCG) by THP-1 cells, bacteria were labeled by incubation with FITC (0.5 mg/ml) in 0.1 M carbonate buffer (pH 9.0) at 37°C for 2 h. Thereafter, FITC-labeled bacteria were washed twice with PBS to remove unbound FITC. Bacteria were opsonized by suspending in 1 ml of RPMI 1640 containing 50% serum and rocked for 30 min at 37°C. Bacteria were then pelleted and resuspended in 1 ml of RPMI 1640 and clumps were disrupted by multiple passages through a 25-gauge needle. THP-1 cells were infected with BCG for 2 h then non-ingested bacteria were removed by extensive washing with PBS followed by trypsinization of the cells. The cells were fixed and bacterial uptake was estimated by flow cytometry (N = 4). (C) Colocalization of Unc119-GFP and Shigella. NIH3T3 cells expressing Unc119-GFP were infected with Shigella 1∶1000 multiplicity for 30 min. The Shigella and nucleus were stained with Hoechst 33258 stain (blue) and false colored as red to show colocalization.
(0.11 MB DOC)

Figure S2 Unc119 interacts with CD44 and activates Fyn. (A) CD44 and alpha5 beta1 are required for Shigella infection. 3T3 cells were treated with anti-CD44 and anti-alpha5 blocking antibodies and their isotype controls for 2 h followed by Shigella infection for 2 h. The cells were processed and the infectivity was measured as described under Figure 1a (N = 6, *P<0.051). (B) Unc119 co-precipitates with CD44 but not with alpha5 beta1. CD44 (upper left panel) and alpha5 (upper right panel) proteins were immunoprecipitated from 3T3 lysates and co-precipitation of Unc119 was checked by western blotting. Rat IgG and mouse IgG3 were used as isotype controls for antibodies against CD44 and alpha5 respectively (N = 3). The lower panel shows immunoprecipitation with Unc119 and western blotting for CD44. Rabbit (Rab) IgG was used as an isotype control. The immunoglobulin heavy chain (IgH) indicates equal protein loading. rUnc119 indicates the position of recombinant Unc119 in the gel. (C) Fyn activation increases during Shigella infection. Control and Unc119 overexpressing cells were serum starved for 24 h then infected with Shigella. Fyn kinase was immunoprecipitated from the infected cells and an auto-phosphorylation assay was performed. The immunoprecipitate was resolved by SDS-PAGE, transferred to a membrane and autoradiographed (upper panel). The membrane was reprobed with an anti-Fyn antibody (lower panel) (N = 3). (D) The effect of Unc119 on Shigella infection is unaltered in SYF cells. Embryonic fibroblasts deficient in Src, Yes and Fyn (SYF) were transfected with siControl and siUnc119 RNA. After 48 h the cells were infected with Shigella and the infectivity was measured (N = 6, *P = 0.042).
(0.10 MB DOC)

Figure S3 Unc119 co-localizes with Arg and Crk in membrane ruffles. 3T3 cells were transfected with the GFP-Unc119 and grown on cover glass over night. The cells were fixed and immunostained using antibodies against Crk (red) and Arg (blue). Lower panels are close-up images of a membrane fold indicated by the arrow (N = 3).
(0.06 MB DOC)

Figure S4 Bright field images of 3T3 cells stably transfected with a control pcDNA3 plasmid or Unc119-pcDNA plasmid. Control cells grow with multiple dendrite-like, sharp cytoplasmic projections and have a relatively small main body. In contrast, Unc119 overexpressing cells show a large and flattened body contour. The cellular projections are smaller in size and show reduced autofluoroescence.
(2.26 MB DOC)

Figure S5 Expression and purification of TAT-GFP, TAT-Unc119 and TAT-Unc119 mutant proteins. (A) The recombinant proteins were expressed as GST fusion proteins and allowed to bind to glutathione agarose beads. The beads were washed and recombinant proteins were cleaved from GST by thrombin. The TAT-Unc119 and TAT-Unc119 mutant proteins were passed through a Sephadex G-50 column. The protein fractions were pooled and the purity of the proteins was checked by polyacrylamide gel electrophoresis followed by Coomassie blue staining. (B) TAT-GFP uptake by 3T3 cells. 3T3 cells were incubated with TAT-GFP (0.1 microM) for 1 h and the uptake was measured by flow cytometry (green plot for cells treated with TAT-GFP). The inset shows the uptake of TAT-GFP observed under a microscope (N = 2). (C) TAT-Unc119 uptake by 3T3 cells. Cells were incubated with recombinant TAT-Unc119 or Unc119 (both at 0.1 microM) for 1 h. The uptake of the recombinant protein was examined by western blotting. Equal protein loading was checked after reprobing the membrane with an anti-tubulin antibody (N = 3). Note TAT-Unc119 migrates slightly slower than the native Unc119. (D) Cells were incubated in 1 microM TAT-Unc119 and its uptake was measured at the indicated time points by western blotting. Cells were incubated with the indicated concentrations of TAT-Unc119 and the uptake was measure after 1 h. (E) 3T3 cells were pretreated with TAT-GFP, TAT-Unc119 or TAT-Unc119 mutant proteins for 1 h followed by a 2 h-infection with Shigella and then infectivity was measured. Results represent the mean±SD of 3 independent experiments in triplicates (*P<0.001).
(1.06 MB DOC)

Figure S6 Induction of Unc119 expression by LPS. BEAS 2B cells were incubated with LPS (50 microgm/ml) for the indicated time period and checked for Unc119. The membranes were reprobed with an anti-tubulin antibody to check for equal protein loading (N = 3).
(0.07 MB DOC)

Figure S7 Unc119 knockdown by shRNA in 3T3 cells. The expression of Unc119 in control vector- and shUnc119 vector- transfected cells was checked after 48 h by western blotting. Equal loading was measured by reprobing the membrane for tubulin (N = 3).
(0.05 MB DOC)

Table S1 Direct binding of Unc119 to Shigella proteins. A sample of Shigella lysate was passed through an Unc119 affinity agarose column (prepared using the Amino-link kit from Pierce Biotechnology, Rockford, IL) and a control agarose column. The unbound proteins were washed with PBS. The bound proteins were eluted with 8 ml 100 mM glycine-HCl (pH 2.5). The eluate (8 ml) was dialyzed (5 mM Tris buffer, pH 7.0) and concentrated by lyophilization. The samples were resuspended in 0.5 ml and equal amounts of sample were resolved on a polyacrylamide gel. The eluate from Unc119 column but not the control column showed three protein bands upon staining with SYPRO Ruby, which were cut and analyzed by LC-MS. This analysis identified three proteins as presented in the Table-1. Existing literature suggests that these are Shigella intracellular proteins and are unlikely to be involved in a direct interaction with Unc119.
(0.04 MB DOC)
