# Supplementary figures and images for: Unc119 Protects from Shigella Infection by Inhibiting the Abl Family Kinases
Source: PLoS One. 2009 Apr 17;4(4):e5211. doi: 10.1371/journal.pone.0005211 (PMC2667249; doi:10.1371/journal.pone.0005211)

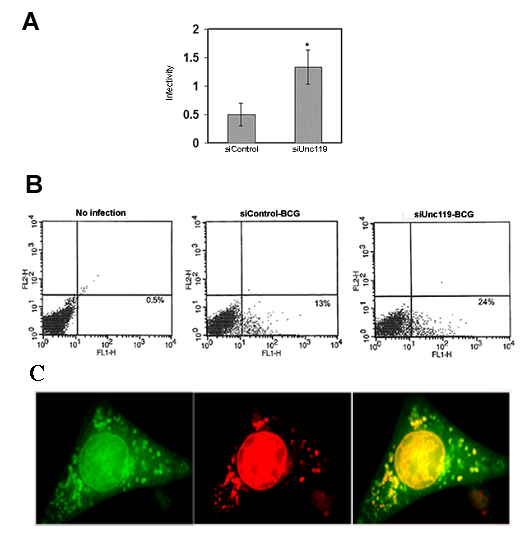

Supplement: Figure S1 — Unc119 knockdown increases the infectivity. A) Infectivity of S. flexneri CVD1203 ( aroA virG) mutant in 3T3 cells. The 3T3 cells were allowed to grow for 48 h followed by infection with Shigella flexneri mutant at a 1∶100 ratio and incubated for 2 h. The cells were washed and the extracellular bacteria were killed with gentamicin. The cells were lysed with 1% Triton X-100 and serial dilutions were plated for colony formation. The bacterial colony forming units were counted and the infectivity was expressed as the number of colony forming units per 10 cells. The results represent mean±SD of six independent experiments. *P = 0.006. (B) Uptake of BCG. To evaluate uptake of Mycobacterium bovis (BCG) by THP-1 cells, bacteria were labeled by incubation with FITC (0.5 mg/ml) in 0.1 M carbonate buffer (pH 9.0) at 37°C for 2 h. Thereafter, FITC-labeled bacteria were washed twice with PBS to remove unbound FITC. Bacteria were opsonized by suspending in 1 ml of RPMI 1640 containing 50% serum and rocked for 30 min at 37°C. Bacteria were then pelleted and resuspended in 1 ml of RPMI 1640 and clumps were disrupted by multiple passages through a 25-gauge needle. THP-1 cells were infected with BCG for 2 h then non-ingested bacteria were removed by extensive washing with PBS followed by trypsinization of the cells. The cells were fixed and bacterial uptake was estimated by flow cytometry (N = 4). (C) Colocalization of Unc119-GFP and Shigella. NIH3T3 cells expressing Unc119-GFP were infected with Shigella 1∶1000 multiplicity for 30 min. The Shigella and nucleus were stained with Hoechst 33258 stain (blue) and false colored as red to show colocalization. (0.11 MB DOC) [file pone.0005211.s001.doc]

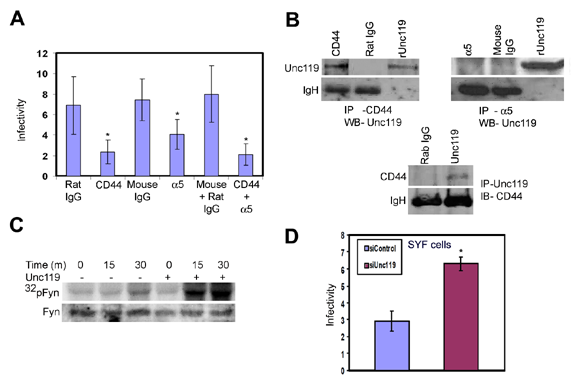

Supplement: Figure S2 — Unc119 interacts with CD44 and activates Fyn. (A) CD44 and alpha5 beta1 are required for Shigella infection. 3T3 cells were treated with anti-CD44 and anti-alpha5 blocking antibodies and their isotype controls for 2 h followed by Shigella infection for 2 h. The cells were processed and the infectivity was measured as described under Figure 1a (N = 6, *P<0.051). (B) Unc119 co-precipitates with CD44 but not with alpha5 beta1. CD44 (upper left panel) and alpha5 (upper right panel) proteins were immunoprecipitated from 3T3 lysates and co-precipitation of Unc119 was checked by western blotting. Rat IgG and mouse IgG3 were used as isotype controls for antibodies against CD44 and alpha5 respectively (N = 3). The lower panel shows immunoprecipitation with Unc119 and western blotting for CD44. Rabbit (Rab) IgG was used as an isotype control. The immunoglobulin heavy chain (IgH) indicates equal protein loading. rUnc119 indicates the position of recombinant Unc119 in the gel. (C) Fyn activation increases during Shigella infection. Control and Unc119 overexpressing cells were serum starved for 24 h then infected with Shigella. Fyn kinase was immunoprecipitated from the infected cells and an auto-phosphorylation assay was performed. The immunoprecipitate was resolved by SDS-PAGE, transferred to a membrane and autoradiographed (upper panel). The membrane was reprobed with an anti-Fyn antibody (lower panel) (N = 3). (D) The effect of Unc119 on Shigella infection is unaltered in SYF cells. Embryonic fibroblasts deficient in Src, Yes and Fyn (SYF) were transfected with siControl and siUnc119 RNA. After 48 h the cells were infected with Shigella and the infectivity was measured (N = 6, *P = 0.042). (0.10 MB DOC) [file pone.0005211.s002.doc]

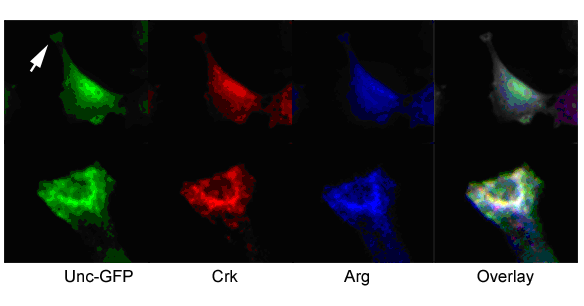

Supplement: Figure S3 — Unc119 co-localizes with Arg and Crk in membrane ruffles. 3T3 cells were transfected with the GFP-Unc119 and grown on cover glass over night. The cells were fixed and immunostained using antibodies against Crk (red) and Arg (blue). Lower panels are close-up images of a membrane fold indicated by the arrow (N = 3). (0.06 MB DOC) [file pone.0005211.s003.doc]

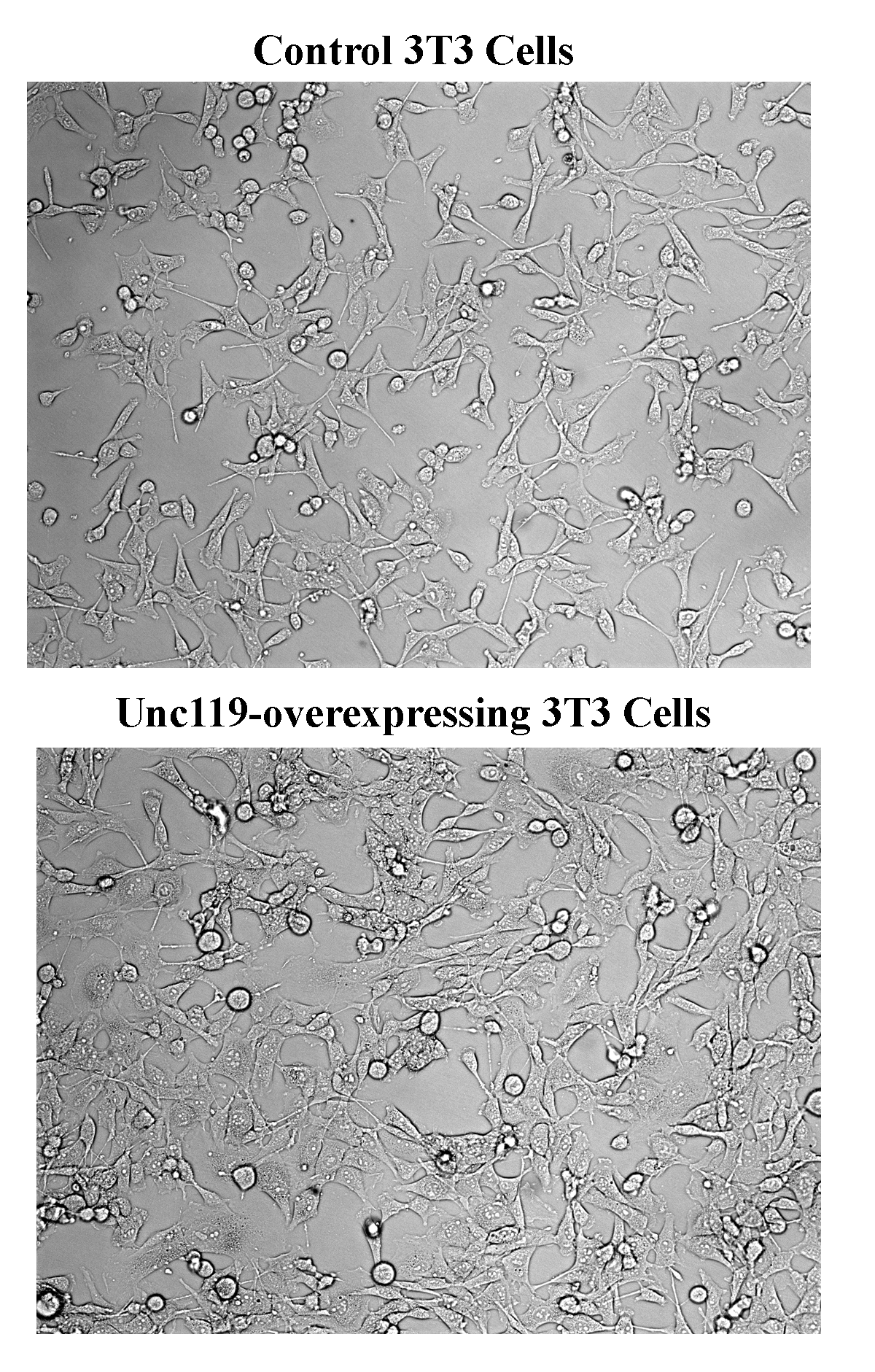

Supplement: Figure S4 — Bright field images of 3T3 cells stably transfected with a control pcDNA3 plasmid or Unc119-pcDNA plasmid. Control cells grow with multiple dendrite-like, sharp cytoplasmic projections and have a relatively small main body. In contrast, Unc119 overexpressing cells show a large and flattened body contour. The cellular projections are smaller in size and show reduced autofluoroescence. (2.26 MB DOC) [file pone.0005211.s004.doc]

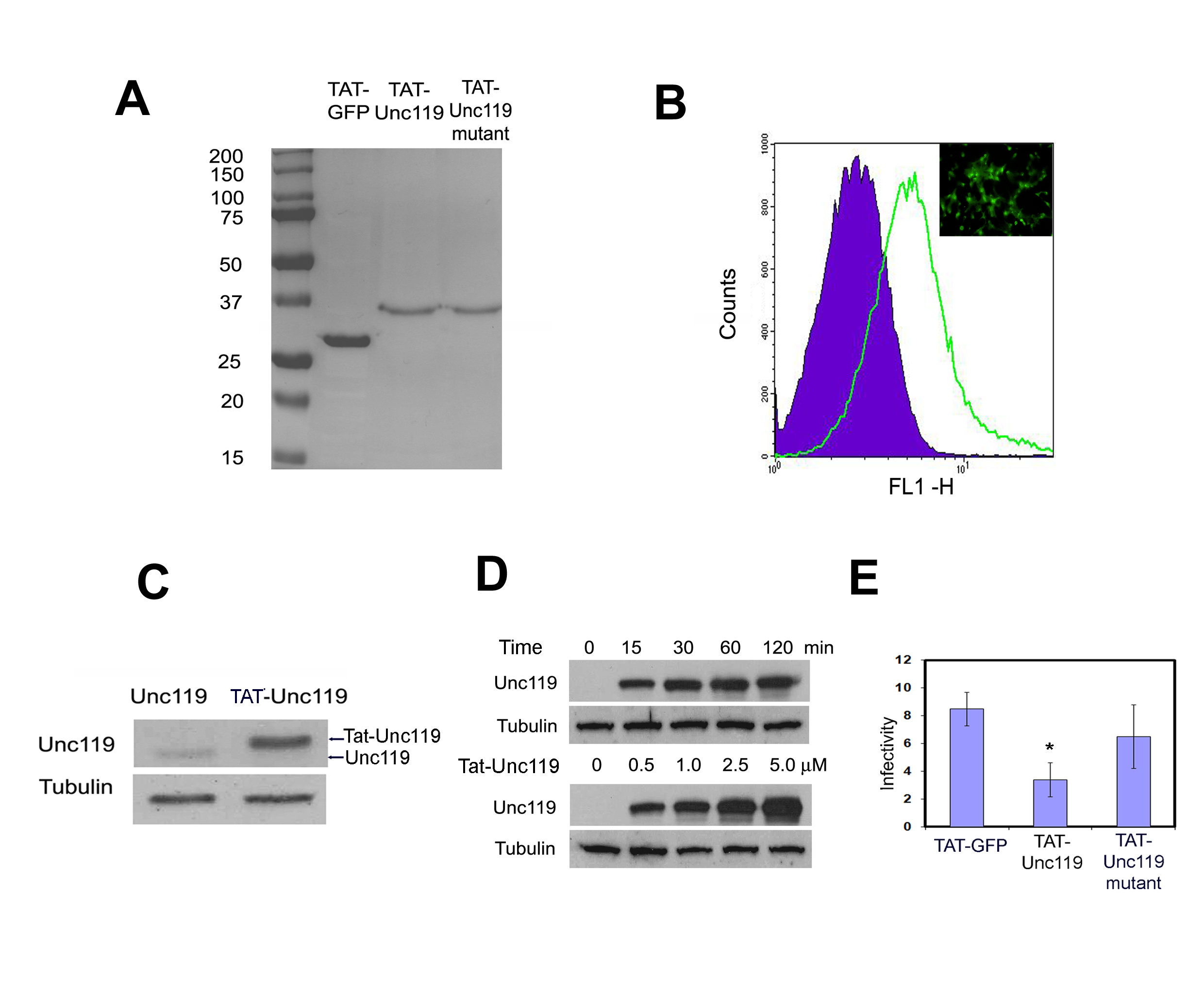

Supplement: Figure S5 — Expression and purification of TAT-GFP, TAT-Unc119 and TAT-Unc119 mutant proteins. (A) The recombinant proteins were expressed as GST fusion proteins and allowed to bind to glutathione agarose beads. The beads were washed and recombinant proteins were cleaved from GST by thrombin. The TAT-Unc119 and TAT-Unc119 mutant proteins were passed through a Sephadex G-50 column. The protein fractions were pooled and the purity of the proteins was checked by polyacrylamide gel electrophoresis followed by Coomassie blue staining. (B) TAT-GFP uptake by 3T3 cells. 3T3 cells were incubated with TAT-GFP (0.1 microM) for 1 h and the uptake was measured by flow cytometry (green plot for cells treated with TAT-GFP). The inset shows the uptake of TAT-GFP observed under a microscope (N = 2). (C) TAT-Unc119 uptake by 3T3 cells. Cells were incubated with recombinant TAT-Unc119 or Unc119 (both at 0.1 microM) for 1 h. The uptake of the recombinant protein was examined by western blotting. Equal protein loading was checked after reprobing the membrane with an anti-tubulin antibody (N = 3). Note TAT-Unc119 migrates slightly slower than the native Unc119. (D) Cells were incubated in 1 microM TAT-Unc119 and its uptake was measured at the indicated time points by western blotting. Cells were incubated with the indicated concentrations of TAT-Unc119 and the uptake was measure after 1 h. (E) 3T3 cells were pretreated with TAT-GFP, TAT-Unc119 or TAT-Unc119 mutant proteins for 1 h followed by a 2 h-infection with Shigella and then infectivity was measured. Results represent the mean±SD of 3 independent experiments in triplicates (*P<0.001). (1.06 MB DOC) [file pone.0005211.s005.doc]

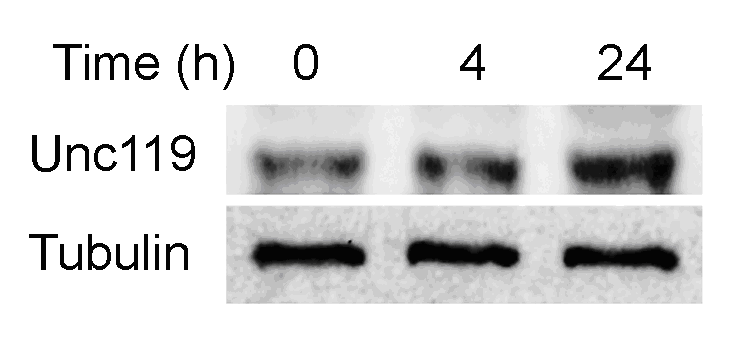

Supplement: Figure S6 — Induction of Unc119 expression by LPS. BEAS 2B cells were incubated with LPS (50 microgm/ml) for the indicated time period and checked for Unc119. The membranes were reprobed with an anti-tubulin antibody to check for equal protein loading (N = 3). (0.07 MB DOC) [file pone.0005211.s006.doc]

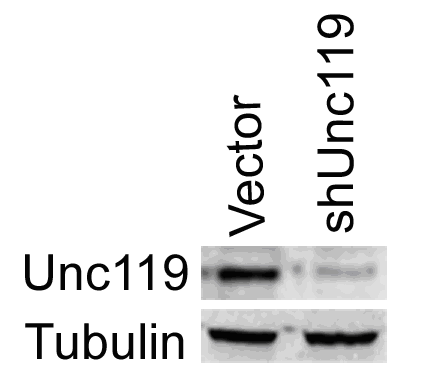

Supplement: Figure S7 — Unc119 knockdown by shRNA in 3T3 cells. The expression of Unc119 in control vector- and shUnc119 vector- transfected cells was checked after 48 h by western blotting. Equal loading was measured by reprobing the membrane for tubulin (N = 3). (0.05 MB DOC) [file pone.0005211.s007.doc]
