# Supplementary material for: Unc119 Protects from Shigella Infection by Inhibiting the Abl Family Kinases
Source: PLoS One. 2009 Apr 17;4(4):e5211. doi: 10.1371/journal.pone.0005211 (PMC2667249; doi:10.1371/journal.pone.0005211)
Supplement: Table S1 — Direct binding of Unc119 to Shigella proteins. A sample of Shigella lysate was passed through an Unc119 affinity agarose column (prepared using the Amino-link kit from Pierce Biotechnology, Rockford, IL) and a control agarose column. The unbound proteins were washed with PBS. The bound proteins were eluted with 8 ml 100 mM glycine-HCl (pH 2.5). The eluate (8 ml) was dialyzed (5 mM Tris buffer, pH 7.0) and concentrated by lyophilization. The samples were resuspended in 0.5 ml and equal amounts of sample were resolved on a polyacrylamide gel. The eluate from Unc119 column but not the control column showed three protein bands upon staining with SYPRO Ruby, which were cut and analyzed by LC-MS. This analysis identified three proteins as presented in the Table-1. Existing literature suggests that these are Shigella intracellular proteins and are unlikely to be involved in a direct interaction with Unc119. (0.04 MB DOC) [file pone.0005211.s008.doc]

| Protein  Name | Distinct peptides | % amino acid  Coverage | MW  (KDa) |
| --- | --- | --- | --- |
| Thioredoxin | 2 | 21 | 14 |
| Elongation factor Tu | 5 | 14 | 43 |
| HSP70 | 6 | 12 | 69 |
